# History of malaria treatment as a predictor of subsequent subclinical parasitaemia: a cross-sectional survey and malaria case records from three villages in Pailin, western Cambodia

**DOI:** 10.1186/s12936-016-1284-8

**Published:** 2016-04-26

**Authors:** Thomas J. Peto, Sabine E. Kloprogge, Rupam Tripura, Chea Nguon, Nou Sanann, Sovann Yok, Chhouen Heng, Cholrawee Promnarate, Jeremy Chalk, Ngak Song, Sue J. Lee, Yoel Lubell, Mehul Dhorda, Mallika Imwong, Nicholas J. White, Lorenz von Seidlein, Arjen Dondorp

**Affiliations:** Mahidol Oxford Tropical Medicine Research Unit, Faculty of Tropical Medicine, Mahidol University, Bangkok, Thailand; Erasmus University of Rotterdam, Rotterdam, The Netherlands; Centre for Tropical Medicine and Global Health, Nuffield Department of Medicine, University of Oxford, Oxford, UK; National Center for Parasitology, Entomology and Malaria Control, Phnom Penh, Cambodia; Family Health International 360, Phnom Penh, Cambodia; Provincial Health Department, Pailin, Cambodia; World Wide Antimalarial Resistance Network, Churchill Hospital, Oxford, UK

**Keywords:** Malaria, *Plasmodium falciparum*, *Plasmodium vivax*, Sub-clinical, Epidemiology, Quantitative polymerase chain reaction, Cambodia

## Abstract

**Background:**

Treatment of the sub-clinical reservoir of malaria, which may maintain transmission, could be an important component of elimination strategies. The reliable detection of asymptomatic infections with low levels of parasitaemia requires high-volume quantitative polymerase chain reaction (uPCR), which is impractical to conduct on a large scale. It is unknown to what extent sub-clinical parasitaemias originate from recent or older clinical episodes. This study explored the association between clinical history of malaria and subsequent sub-clinical parasitaemia.

**Methods:**

In June 2013 a cross-sectional survey was conducted in three villages in Pailin, western Cambodia. Demographic and epidemiological data and blood samples were collected. Blood was tested for malaria by high-volume qPCR. Positive samples were analysed by nested PCR to determine the *Plasmodium* species. To identify previous episodes of malaria, case records were collected from village malaria workers and local health facilities and linked to study participants.

**Results:**

Among 1343 participants, 40/122 (32.8 %) with a history of clinical malaria were parasitaemic during the cross-sectional survey, compared to 172/1221 (14.1 %) without this history (p < 0.001). Among the 212 parasitaemic participants in the survey, 40 (18.9 %) had a history of clinical malaria, compared to 87 out of 1131 (7.7 %) parasite-negative participants; p < 0.001, adjusted OR 3.3 (95 % CI; 2.1–5.1). A history of *Plasmodium vivax* was associated with sub-clinical *P. vivax* parasitaemia in the survey (p < 0.001), but this association was not seen with *Plasmodium falciparum* (p = 0.253); only three participants had both *P. falciparum* parasites in the survey and a clinical history of *P. falciparum*.

**Conclusions:**

A clinical episode of vivax malaria was associated with subsequent sub-clinical parasitaemia. Treatment of *P. vivax* with artemisinin-based combination therapy without primaquine often resulted in recurrent episodes. Targeting individuals with a history of clinical malaria will be insufficient to eliminate the sub-clinical reservoir as they constitute a minority of parasitaemias.

## Background

Pailin Province in western Cambodia was the first region where artemisinin-resistant *Plasmodium falciparum* was reported [1, 2]. Subsequently, artemisinin-resistant parasites have been found on the Thai-Myanmar border and recently in other parts of Southeast Asia [3]. In the past, *P. falciparum* strains resistant to chloroquine, sulfadoxine–pyrimethamine and mefloquine emerged in western Cambodia and spread across Southeast Asia to Africa [3–5]. Artemisinin-based therapy is globally the most effective of anti-malarials and first-line drugs for malaria. Artemisinin resistance poses a serious threat to malaria control [6]. There is an emerging consensus that preventing the spread of artemisinin-resistant malaria requires the elimination of all *P. falciparum* in areas of artemisinin resistance [7–9].

Malaria incidence in Pailin Province has dropped markedly over the last decade [10]. The incidence rate of malaria declined from 158/1000 cases per year in 2004 to 13.1/1000 in 2011 (Provincial Health Department, Pailin). Malaria control has improved over this period, including early treatment of clinical cases by village malaria workers (VMWs), health education to raise awareness about malaria and the distribution of long-lasting, insecticide-treated nets (LLINs) [10]. There have been multiple first-line malaria treatments in Cambodia over the past decade due to the rapid emergence and spread of resistant *P. falciparum* parasites [6, 11] (Appendix: Table 7). Dihydroartemisinin-piperaquine is currently the first-line treatment for both uncomplicated *P. falciparum* and *Plasmodium vivax* in western Cambodia (Cambodian National Malaria Control Programme treatment guidelines).

Recent studies demonstrate a large reservoir of sub-microscopic malaria parasites in areas of low transmission in Southeast Asia [12, 13], including Pailin [14]. These sub-clinical levels of parasitaemia are likely to be an important source of transmission, and to be responsible for the surge in clinical malaria at the start of the transmission season when vector capacity increases [12]. The estimate of malaria prevalence is dependent on the sensitivity of the detection method used. Parasite densities in asymptomatic carriers can often only be detected by polymerase chain reaction (PCR) techniques, which are expensive and time consuming [15]. A better understanding of the relationship between clinical malaria incidence and asymptomatic malaria parasite prevalence is useful for planning cost-efficient surveillance strategies. If a large proportion of sub-clinical parasitaemias originate from previously treated malaria cases, then people with a history of malaria could be targeted as part of an elimination programme, and incident clinical cases of malaria could become a target for enhanced follow-up to achieve radical cure. Moreover, knowledge of the relationship between clinical and sub-clinical parasitaemia might allow an estimation of sub-clinical reservoirs. This study investigated to what extent sub-clinical parasitaemia was associated with past clinical episodes of malaria. The objective of this study was to describe and assess the association between symptomatic malaria and subsequent sub-clinical parasitaemia.

## Methods

### Sub-clinical parasitaemia

A cross-sectional survey was conducted in June 2013 in three villages in Pailin Province in western Cambodia to estimate the prevalence of sub-clinical malaria parasitaemia. The neighbouring villages of Krachap Leu (KL), O Kting (OK) and Phnom Dambang (PDB) were selected because local treatment records indicated the highest malaria incidence within Pailin Province in 2012. Public meetings were held to inform the villages about the study and individual, written, informed consent was obtained from all participants or their guardians. Basic demographic, anthropometric and health history data were collected from everyone above 6 months of age who was in the villages during the 3 weeks of the study.

### Laboratory procedures

Three ml venous blood samples from participants ≥5 years and 500 µl from children from 6 months up to 5 years old were collected in EDTA tubes. Samples were stored in a cool box until transport to the Pailin Referral Hospital laboratory where they were centrifuged to separate plasma, buffy coat and packed red blood cells (pRBC). Each sample was labelled with a barcode to ensure blinding, then stored at −80 °C until transport on dry ice to the Mahidol University Faculty of Tropical Medicine molecular laboratory in Bangkok, Thailand.

Batches of 500 µl (~100 µl for those from 6 months up to 5 years) of pRBC were thawed and high-volume ultra-sensitive qPCR (uPCR) was performed to detect malaria parasites. The uPCR technique (Qiagen, Germany) has a limit of accurate detection of 22 parasites/ml. [16] Parasite density was quantified using the Rotor-Gene Q series software v 2.0.2. Samples testing positive for *Plasmodium* were then analysed by nested PCR to determine species [15, 16].

### Symptomatic malaria

In each of the study villages VMWs used rapid diagnostic tests (RDTs) and provided free treatment for malaria. They were supported by the mobile malaria workers (MMWs) who provided the same services and were responsible for migrants. For each patient, demographic information (including whether resident or migrant) and the diagnosis and treatment given were recorded. The villages were in the catchment area of one peripheral health facility, Krachap Health Centre, which used RDTs to diagnose malaria. Severe cases of malaria were managed by Pailin Referral Hospital, the only public hospital in Pailin. The hospital used microscopy for diagnoses. All available malaria case records relating to treatment of study participants prior to the June 2013 survey were collected from VMWs, MMWs, the Health Centre, and the Hospital. The records covered the period June 2005 to 2013.

Case records were reviewed by two members of staff and then linked carefully to the survey participants by matching names, ages, gender, and where relevant, guardian. Correct linkage was confirmed by interview with the participant, their guardian, and/or the VMW or MMW who treated them. Linkage was finalized while blind to the participants’ parasitaemia status in the June 2013 survey. Participants from the cross-sectional survey who responded that they were temporarily resident (and, therefore, would not reliably appear in local treatment records if treated for malaria prior to the survey) were excluded from the analysis.

### Definitions

A case history of malaria was defined as having had clinical malaria at some point prior to the cross-sectional survey as identified from the case records. A self-reported history of malaria was defined by the survey question “have you ever had malaria in the past?” and this category could include people who did not otherwise appear in available malaria treatment records. Participants who tested positive for malaria parasites by qPCR in the cross-sectional survey were defined as having sub-clinical parasitaemia. Each participant was asked whether they were temporarily (not a member of a permanent household) or permanently living in the village.

### Statistical analysis

Groups were compared using a Student’s *t* test, the Chi squared test or Fisher’s exact test, as appropriate. The analysis was repeated using the proportion of all the survey participants who self-reported a history of malaria. The association between a case history of malaria and subsequent sub-clinical parasitaemia was quantified using logistic regression. All variables that were significant in the univariate analysis at p < 0.05 were included in a logistic regression model with sub-clinical parasitaemia (Y/N) as the outcome. Using a stepwise approach, risk factors that were significant at p < 0.05 were identified and retained in the final model. Data were analysed using STATA, version 13.1 (StataCorp LP, College Station, TX, USA).

### Ethical approval

Ethics approval was obtained from the Oxford Tropical Research Ethics Committee (OXTREC; 1015-13), National Ethics Committee for Health Research Cambodia (0029 NECHR), and the study was registered on clinicaltrials.gov (NCT01872702).

## Results

### Cross-sectional survey and case histories

**Fig. 1:**
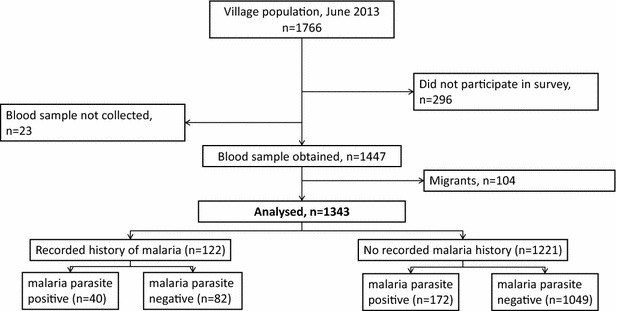
Study consort diagram

**Fig. 2:**
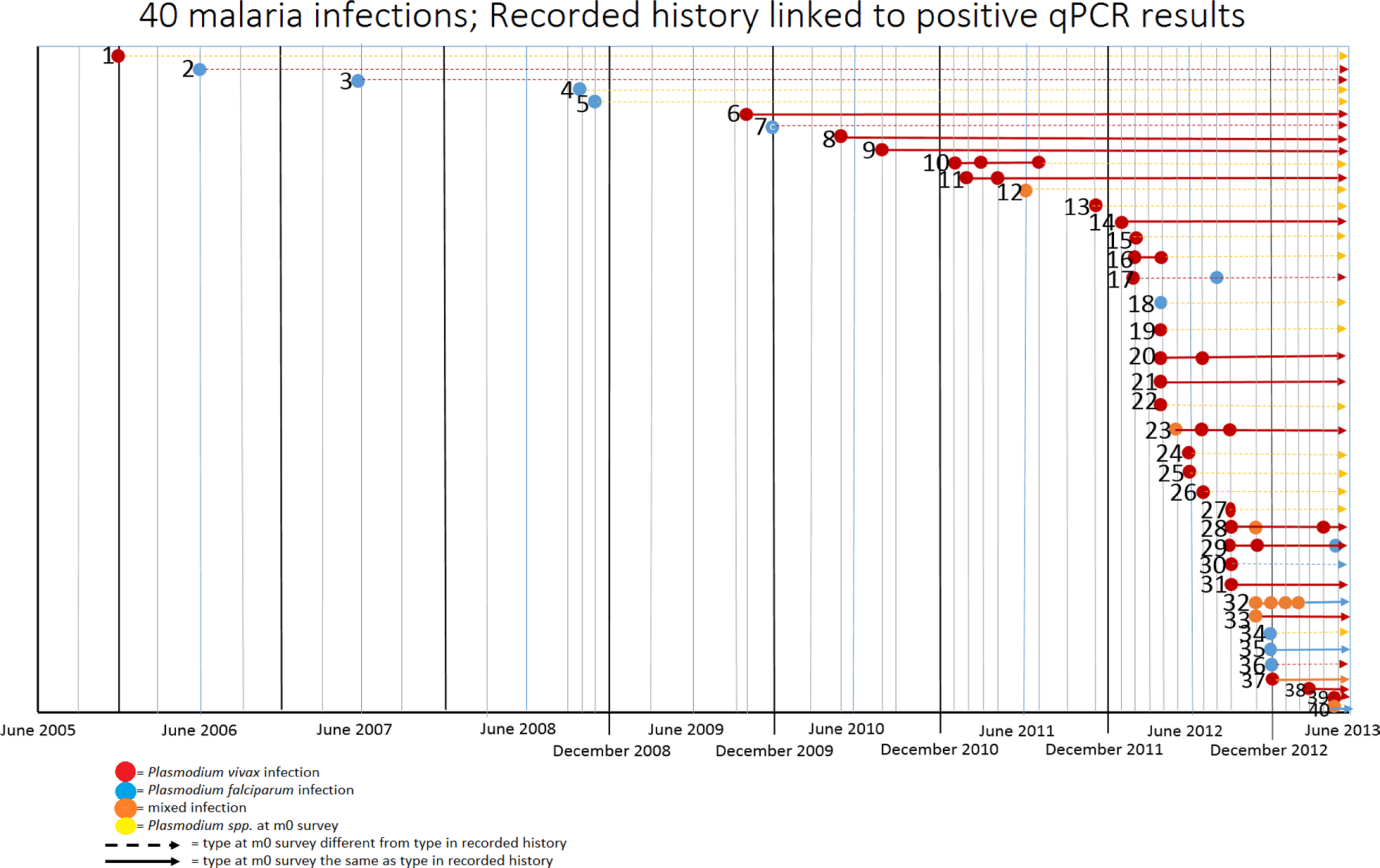
Time since treatment among those who were parasitaemic in the June 2013 survey and who also appeared in local health records as having been previously treated for malaria

In April 2013, the census population of the three villages was 1758. In June 2013, malaria test results and surveys were obtained from 1447 villagers (82.3 %). One-hundred and four migrants were excluded, leaving 1343 participants for the main analysis (Fig. 1). A total of 410 malaria case records were collected: 258 from VMWs, 96 from the Health Centre, 45 from the Referral Hospital (of which 32 were from clinical studies), and 11 from MMWs, dating from 2005 to June 2013. Of 410 case records, 122 (29.8 %) were linked to surveyed villagers. The quality of case records improved from 2011 onwards after the strengthening of the VMW programme; 81/122 (66.4 %) of all the case records linked to survey participants come from 2011 onwards (Fig. 2).

### Description of the study population

There were 212/1343 (15.8 %) participants with sub-clinical parasitaemia at the time of the survey, and 122/1343 (9.1 %) had a case history of malaria. Seventeen of 104 (16.3 %) of the migrants surveyed had sub-clinical parasitaemia. Those who were aged >15 years, male or from Phnom Dambang village were more likely to have a history of malaria (Table 1) and also sub-clinical parasitaemia during the June 2013 survey [17, 18].

**Table 1:** Baseline characteristics of participants from the June 2013 cross-sectional survey, by recorded history of clinical malaria (2005–13)

	Case record of clinical malaria n = 122 (9 %)	No case record of clinical malaria n = 1221 (91 %)	All villagers n = 1343	P value*
Gender (male)	81 (66 %)	597 (49 %)	687 (51 %)	<0.001
Age (years; mean)	26.8	24.1	24.3	0.107
Age groups (years)				
0–4.9	2 (2 %)	161 (13 %)	163 (12 %)	
5.0–14.9	31 (25 %)	334 (27 %)	365 (27 %)	
15.0–44.9	70 (57 %)	511 (42 %)	581 (43 %)	
≥45.0	19 (16 %)	215 (18 %)	234 (17 %)	<0.001
Village				
Krachap Leu	30 (25 %)	436 (36 %)	466 (35 %)	
O Kting	38 (31 %)	240 (20 %)	278 (21 %)	
Phnom Dambang	54 (44 %)	545 (45 %)	599 (45 %)	0.004
Occupation				
Child or student	35 (29 %)	525 (43 %)	560 (42 %)	
Farmer other land	20 (16 %)	102 (8 %)	122 (9 %)	
Farmer own land	56 (46 %)	443 (36 %)	499 (37 %)	
Farm labourer	8 (7 %)	101 (8 %)	109 (8 %)	
Other	3 (3 %)	50 (4 %)	53 (4 %)	0.002
Recent forest visit	11 (9 %)	38 (3 %)	49 (4 %)	0.001
Recent travel history	23 (19 %)	243 (20 %)	266 (20 %)	0.782
Bed net use				
Regular	118 (97 %)	1193 (98 %)	1311 (98 %)	
Irregular	4 (3 %)	28 (2 %)	32 (2 %)	0.496
Low grade fever ≥37.5 °C	4 (3 %)	69 (6 %)	73 (5 %)	0.270
Fever, by self-report at survey	18 (15 %)	148 (12 %)	166 (12 %)	0.401
Illness, self-report at survey	32 (26 %)	371 (30 %)	403 (30 %)	0.334

* Comparing those with and without a clinical history of malaria

Recent travel to a forest and being a farmer with their own land were associated with a history of malaria (p < 0.001 and p = 0.002, respectively), but not with sub-clinical parasitaemia (p = 0.614 and p = 0.068, respectively). Mean age, travel history, use of bed nets, history of fever, history of illness and low-grade fever (temperature 37.5 °C or higher) were not associated with a history of malaria.

### Main outcome

**Fig. 3:**
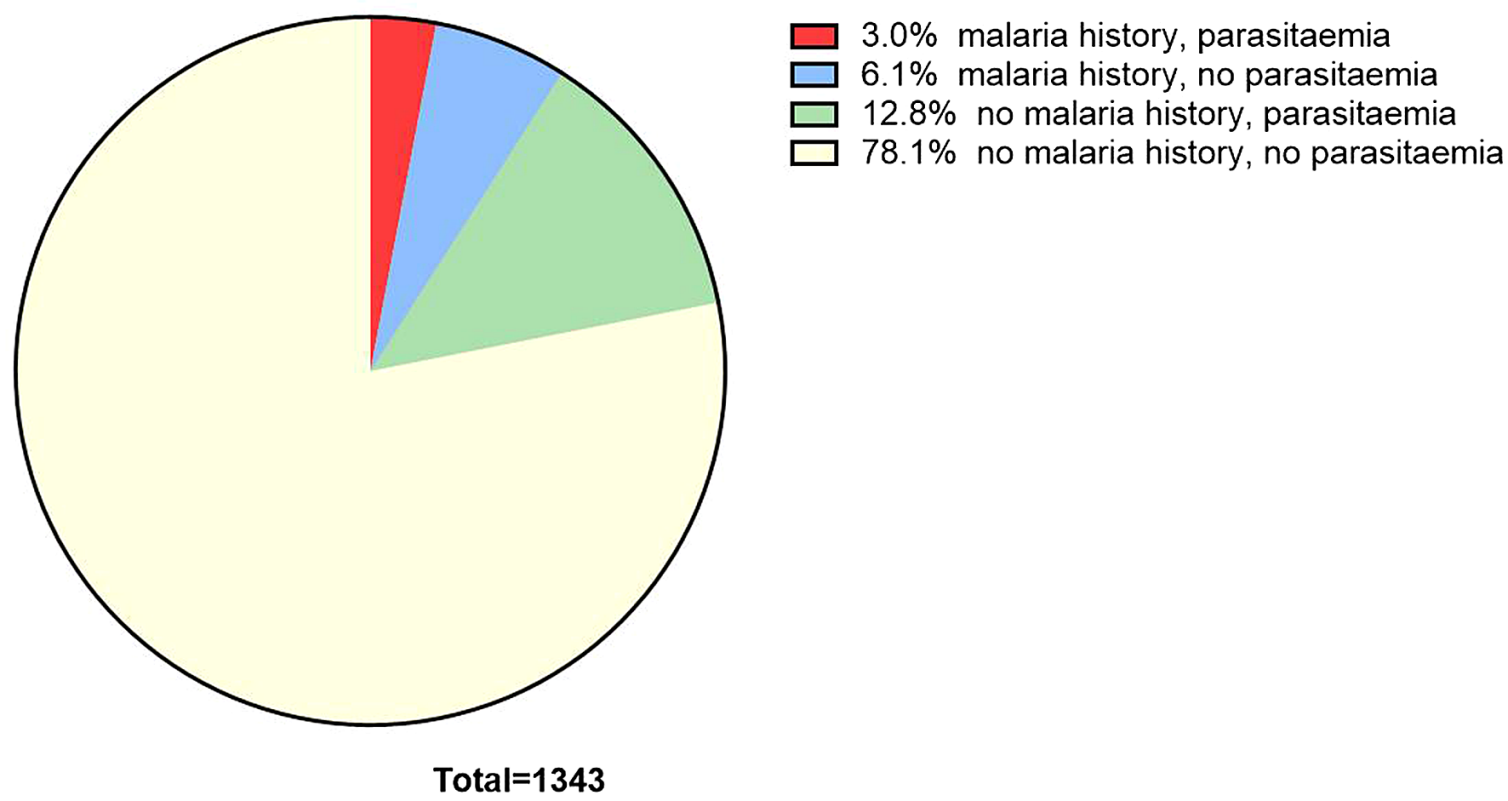
Recorded history of clinical malaria 2005–13 and subsequent parasitaemia in the June 2013 cross-sectional survey

The prevalence of any parasitaemia during the survey was higher (40/122 (32.8 %) among those with a recorded history of malaria when compared to those without [172/1221 (14.1 %), p < 0.001] (Table 2). The majority of people did not have sub-clinical parasitaemia in the survey and did not appear in local treatment records (Fig. 3). Among those with sub-clinical parasitaemia in the survey, the odds ratio for a recorded history of malaria was 3.0 (95 % CI; 2.0–4.5); after adjustment for age group, gender and village, the OR increased to 3.3 (95 % CI; 2.1–5.1).

**Table 2:** Association between a case history of malaria in local health records (2005–13) and sub-clinical *Plasmodium* parasitaemia in the June 2013 cross-sectional survey

	Case history of malaria	No case history of malaria	Total
Subclinical parasitaemia	40 (33 %)	172 (14 %)	212 (16 %)
No subclinical parasitaemia	82 (67 %)	1049 (86 %)	1131 (84 %)
Total	122 (100 %)	1221 (100 %)	1343 (100 %)

p < 0.001

In 140/212 sub-clinical parasitaemias, the species of malaria could not be determined by nested PCR. Where known, the most common species of sub-clinical parasitaemia was *P. falciparum* in those without a recorded history of malaria and *P. vivax* in those who did have a case history of malaria (Table 3). *P. vivax* and mixed *P. vivax* infections in malaria case records were strongly associated with *P. vivax* and mixed *P. vivax* parasitaemia during the survey; p < 0.001 (Table 4). This association was not found between *P. falciparum* and mixed *P. falciparum* case records and *P. falciparum* and mixed *P. falciparum* parasitaemia during the survey, p = 0.253 (Table 5). Only three participants had a history of *P. falciparum* and were positive for *P. falciparum* in the survey. All of these cases occurred within the 6 months prior to the survey (Fig. 2). No association was found between the number of recorded malaria episodes and sub-clinical parasitaemia p = 0.300.

**Table 3:** *Plasmodium* species among sub-clinical parasitaemias in the June 2013 cross-sectional survey, by a case history of malaria in local health records (2005–13)

Plasmodium species	Case history of malaria	No case history of malaria	Total
*P. falciparum*	0	0	0
Mixed *P. vivax P. falciparum*	5 (22 %)	18 (78 %)	23
*P. vivax*	1 (25 %)	3 (75 %)	4
Plasmodium, species not defined	18 (40 %) 16 (11 %)	27 (60 %) 124 (89 %)	45 140
Total	40 (19 %)	172 (81 %)	212 (100 %)

**Table 4:** *Plasmodium vivax* sub-clinical parasitaemia in the June 2013 cross-sectional survey by a case history of malaria in local health records (2005–13)

	*P. vivax* malaria case history	No *P. vivax* malaria case history	Total
*P.vivax* subclinical parasitaemia	15 (18 %)	34 (3 %)	49 (4 %)
No *P.vivax* subclinical parasitaemia	70 (82 %)	1224 (97 %)	1294 (96 %)
Total	85 (100 %)	1258 (100 %)	1343

p < 0.001

**Table 5:** *Plasmodium falciparum* sub-clinical parasitaemia in the June 2013 cross-sectional survey by a case history of malaria in local health records (2005–13)

	*P. falciparum* malaria case history	No *P. falciparum* malaria case history	Total
*P.falciparum* subclinical parasitaemia	3 (4 %)	24 (2 %)	27 (2 %)
No *P.falciparum* subclinical parasitaemia	77 (96 %)	1239 (98 %)	1316 (98 %)
Total	80 (100 %)	1263 (100 %)	1343

p = 0.253

### Self-reported histories of clinical malaria

Of the participants who were linked to a malaria case record, 98.4 % (120/122) also self-reported having had malaria prior to the survey; 77.7 % (418/538) of those who self-reported having had malaria did not appear in the available malaria case records. Among these 418 participants who reported malaria (but for whom no case record was found), there was a statistically significant association with sub-clinical parasitaemia during the survey [OR 1.5 (95 % CI; 1.1–2.0) p = 0.023, Table 6]. This association was statistically non-significant when adjusted for age, gender and village (p = 0.100).

**Table 6:** Sub-clinical *Plasmodium* parasitaemia and a self-reported history of clinical malaria recorded in the cross-sectional survey, among those who did not have a case history of malaria in local health records

	Self-reported history of malaria	No self-reported history of malaria	Total
Subclinical parasitaemia	72 (17 %)	100 (12 %)	172 (14 %)
No subclinical parasitaemia	346 (83 %)	703 (88 %)	1049 (86 %)
Total	418 (100 %)	803 (100 %)	1221

p = 0.023

**Table 7:** Provincial Health Department guidelines for first line antimalarial treatment in Pailin province

Drug policy period	*P.* *falciparum*	*P.* *vivax*
2002–2003	Artesunate-Mefloquine	Chloroquine
2003–2010	DHA-PPQ	Chloroquine
2010–2012	DHA-PPQ	DHA-PPQ
2012–2014	Atovaquone-Proguanil	DHA-PPQ
2014	DHA-PPQ	DHA-PPQ

## Discussion

In Pailin, sub-clinical parasitaemia with any malaria species was three times more common among those with a history of malaria than those without. This may be explained chiefly by the persistence or relapse of *P. vivax* parasites among previously treated cases, which suggests that treatment of *P. vivax* without primaquine often fails to result in radical cure. There was no clear association between the case records of *P. falciparum* and subsequent sub-clinical *P. falciparum* parasitaemia in the survey. Only three participants had both a case record of *P. falciparum* and *P. falciparum* parasitaemia in the survey, and all had been infected within the previous 6 months.

The relationship between malaria incidence and the sub-clinical prevalence of parasitaemia is currently poorly defined. There were 55 malaria cases of all species recorded among residents of the study villages in 2012 (an annual parasite incidence of approximately 4.1 %). The parasite prevalence during the uPCR survey in June 2013 was 15.8 %, suggesting an approximately fourfold ratio. This information may be of relevance to elimination efforts as it could inform areas to target, but will require confirmation from other studies in other geographic areas. The origin of the large number of sub-clinical parasitaemias among those with no recorded or self-reported history of malaria remains unknown, as does the duration and potential infectiousness of sub-clinical *P. falciparum* parasitaemias in this population. The persistence of sub-clinical *P. vivax* parasitaemia among previously treated cases highlights again the critical importance of 8-aminoquinolines for the complete cure. 8-aminoquinolines are currently not used widely in Cambodia due to concerns about haemolytic adverse effects among people with glucose-6-phosphate dehydrogenase deficiency.

### Limitations

Prior to June 2011 VMW treatment records were not systematically collected. This missing information implies that this study may underestimate the association between treatment and sub-clinical parasitaemia. There is considerable seasonal migration in Pailin and case records of residents who received malaria treatment outside of the study area were not collected. Records could not be obtained from the private sector, where malaria treatment was common practice until it was banned in 2012, and frequently involved the use of ineffective anti-malarial drugs [19]. The sources from which case records were available generally followed the national guidelines for treatment of malaria, but the paucity of these data meant associations between specific anti-malarial drugs and subsequent sub-clinical parasitaemia could not be analysed. Data from the follow-up of clinical cases to assess efficacy at day 28 would have been informative, but were also unavailable. Lastly, parasite densities of sub-clinical parasitaemia may be below the detection threshold of uPCR and were missed in the cross-sectional design.

## Conclusions

Clinical episodes of vivax malaria were associated with subsequent sub-clinical parasitaemia. Treatment of *P. vivax* with artemisinin-based combination therapy without primaquine often resulted in recurrent episodes. The at least threefold risk of sub-clinical parasitaemia among those with a recorded case history of malaria suggests an easy-to-identify, high-risk population. Other high-risk groups, such as family members of malaria cases or forest workers, have been identified by previous studies [14]. None of these groups constitute the majority of infections within the village population and many people harbour parasites but have no particular risk factors [17]. Therefore, to eliminate all malarias from an area, treatment with the aim of interrupting transmission should target the entire community.

## Appendix

National Treatment Guidelines, Cambodia National Malaria Control Programme (see Table 7).

## Abbreviations

ACT: artemisinin combination therapy
CNM: Cambodian National Malaria Control Programme
DHA-PQP: dihydroartemisinin-piperaquine
uPCR: high-volume ultra-sensitive qPCR
LLIN: long-lasting insecticide-treated nets
MMW: mobile malaria worker
MORU: Mahidol-Oxford Tropical Medicine Research Unit
PCR: polymerase chain reaction
PHD: Provincial Health Department
pRBC: packed red blood cells
qPCR: quantitative polymerase chain reaction
RDT: rapid diagnostic test
TME: targeted malaria elimination
VMW: village malaria worker
